# The association of 20 short tandem repeat loci of autosomal chromosome with male schizophrenia

**DOI:** 10.1002/brb3.2637

**Published:** 2022-06-08

**Authors:** Chun Yang, Huajie Ba, Huihui Zou, Xianju Zhou

**Affiliations:** ^1^ The 904th Hospital (Changzhou Branch) of Joint Logistic Support Force of Chinese People's Liberation Army Changzhou Jiangsu Province China; ^2^ DNA Laboratory Public Security Bureau of Changzhou Changzhou Jiangsu Province China; ^3^ Special Medical Service Center, Neuroscience Center, Integrated Hospital of Traditional Chinese Medicine Southern Medical University Guangzhou Guangdong China

**Keywords:** allele, autosomal chromosome, haplotype, schizophrenia, short tandem repeats

## Abstract

**Introduction:**

Schizophrenia's heritability and familial transmission have been known for several decades. The male‐specific Y chromosome plays an important role in schizophrenia. Short tandem repeats (STRs)have been recognized as risk genes in the development of schizophrenia. Here, we investigated the association between male schizophrenia and Y‐chromosomal STRs loci.

**Methods:**

We recruited 355 patients with schizophrenia and 473 healthy males for physical examination and amplified them with a PowerPlex 21 System fluorescence‐labeled composite amplification System. Then, the resultant products were separated by electrophoresis and further detected. Finally, differences in allele and genotype frequency distributions of STR loci were observed.

**Results:**

Our results showed that all 20 STR loci were in accordance with Hardy–Weinberg's law (*p* > .05). There were statistically significant differences in alleles of D13S317 and D5S818 loci and genotype frequency distribution between the two groups (alleles: *p* = .039, *p* = .022, respectively; genotype: *p* = .0004, *p* = .011, respectively). However, there was no difference in the other autosomal 18 STR loci between the two groups (*p* > .05). Univariate analysis showed that the frequency distribution differences of allele 11 and genotype 10‐11 at the D13S317 locus between the two groups were significant (compared to the controls, *p* = 0.005, odds ratio (OR) = 1.37, 95%b confidence interval (CI) = 1.10–1.71, compared to the controls, *p* = .0000002, OR = 3.92, 95% CI = 2.27–6.77, respectively). The frequency distribution differences of allele 7 and genotype 7‐10 at D5S818 between the two groups were significant (compared to the controls, *p* = .0006, OR = 3.42, 95% CI = 1.63–7.16, compared to the controls, *p* = .0011, OR = 8.24, 95% CI = 1.83–37.05, respectively).

**Conclusion:**

Polymorphisms of the D13S317 and D5S818 loci may be predisposing factors for schizophrenia.

## Graphical abstract

To explore the relationship of short tandem repeat (STR) loi with male schizophrenia, allelic and haplotypic distribution frequencies of 20 STRs on autosomal chromosomes were compared between schizophrenia patients and normal subjects in a population of Chinese males. Polymorphisms of the D13S317 and D5S818 loci may be associated with the onset of schizophrenia in males. Allele 11 and genotype 10‐11 of the D13S317 locus and allele 7 and genotype 7‐10 of the D5S818 locus may predispose patients to male schizophrenia.

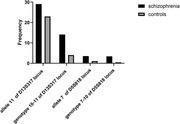

## 1 INTRODUCTION

Schizophrenia is a psychiatric complex affecting about 1% population of the world and is one of the top 10 causes of disability globally (Fazel et al., 2009). Schizophrenia has a remarkable impact on schizophrenia patients, their families, and society (Singh et al., 2012). Above 50% of admitted schizophrenia patients have intermittent and prolonged mental problems, and about 20% show disabilities as well as chronic symptoms. The exact causes of psychosis are unknown, leading to a lack of effective treatments (Marder & Cannon, 2019; Winship et al., 2019). Therefore, we explored the association between the short tandem repeat (STR) locus (Bär et al., 1997) and male schizophrenia and screened out the corresponding chromosomal regions associated with schizophrenia, which has an important significance for further searching for schizophrenic‐related genes.

Schizophrenia is a complex cognitive and behavioral disorder influenced by both genetics and the environment (McCutcheon et al., 2020). For example, early environmental exposure and the influence of genetic factors on brain development may increase the risk of schizophrenia. Small abnormalities in brain structures, such as gray matter and white matter (Meyer‐Lindenberg & Tost, 2014), the dysfunction of normal synaptic function and abnormal activation of stress‐related signaling pathways (including inflammatory and oxidative stress) have been verified (Owen et al., 2016).

There is accumulating evidence that schizophrenia has a profound genetic basis. It was reported that (1) a few putative susceptibility loci, such as 5q, 6p, 8p, 10p, 13q, and 22q, were associated with schizophrenia and (2) a rare variant was associated with schizophrenia. A copy number variation was observed at 8 loci, that is, 1q21.1, 2p16.3, 3q29, 7q11.2, 15q13.3, distal and proximal 16p11.2, and 22q11.2 (Birnbaum & Weinberger, 2017). Epidemiological studies revealed that schizophrenia is affected by genetic factors. Previous studies have linked STR loci with many diseases and abnormal behaviors. A growing body of evidence suggests that STR plays an important role in complex traits. STR has been shown to cause Huntington's disease and genetic ataxia (Fotsing et al., 2019). Chun and colleagues (Chun & Ba, 2012; Chun et al., 2014) also showed that violent aggression in non‐mental diseases was associated with STR loci (TPOX, TH01, D2S1338, D19S433, etc.). Therefore, exploring the correlation between STR loci and male schizophrenia can help screen out corresponding chromosomal regions associated with male schizophrenia, which is of great significance for further searching for genes related to aggression in schizophrenia.

Short repeats, consisting of consecutive 1–6 base pairs, are a source of great genetic variation (Fotsing et al., 2019). STR is composed of tandem repeating DNA units scattered throughout the genome with highly heterozygous and evolutionary information (Luan et al., 2020). Genetic polymorphism analysis of STR loci has been utilized worldwide in many fields, including paternity detection, genetic mapping, disease linkage investigation, evolutionary biology, and the genetics of population. Our previous data showed that two autosomal STRs (TH01 and TPOX) were related to impulsive aggression, and the two other autosomal STRs (Penta D and Penta E) were involved in initiative aggression (Wyner et al., 2020; C. Yang et al., 2018). However, so far there have been no data to suggest the correlation between STRs on the autosomal chromosomes and male schizophrenia. Therefore, in the present study, 20 STRs on autosomal chromosomes were genotyped in male schizophrenia patients and normal male controls to examine whether some STR loci on autosomal chromosomes were correlated with male schizophrenia.

## 2 MATERIALS AND METHODS

### 2.1 Participants

This study finally recruited 355 male subjects suffering from schizophrenia (*n* = 355) between 2010 and 2015. The average age of these subjects was 30.6 ± 10.4 years. A total of 26, 139, 120, and 68 subjects received different levels of education, including illiterate level (2), primary school (26), junior school (139), high school (120), and higher education (68). Among them, marital status was as follows: 128 were married, 196 were unmarried, and 31 were divorced. The course of the disease was 23.5 ± 8.3 years. These subjects lacked a history of neurological disorders, other organic disorders, or mental retardation. Moreover, no subjects suggested a history of substance use or brain injury. The male controls included 473 age‐matched healthy males, with an average age of 29.7 ± 9.6 years, who participated in the physical examination and were recruited from People's Liberation Army Joint Logistic Support Force 904th Hospital in Changzhou, China. The distribution of their education levels was 49 at the primary school level, 161 at the junior school level, 172 at the high school level, and 91 at the higher education level. Among them, marital status: 181, married; 263, unmarried; 29, divorced. All subjects were Han Chinese males living in Jiangsu Province, with no relatives of each other. No significant difference was found in age (*t* = 1.29, *p* = .198), marital status (χ ^2^ = 2.17, *p* = .339), or educational level (χ ^2^ = 6.57, *p* = .160) between the two groups. This study was approved by the Ethics Committee of People's Liberation Army Joint Logistic Support Force 904th Hospital, and all experiments were carried out in accordance with approved guidelines.

### 2.2 STR locus selection and genotyping

DNA was extracted from peripheral blood using the conventional Chelex‐100 extraction method (Schneider, 1997; Walsh et al., 2013). The 20 STR loci D3S1368 (3p21.31), D1S1656 (1q42), D6S1043(6q15), D13S317(13q31.1), Penta E (15q26.2), D16S639(16q24.1), D18S51(18q21.33), D2S1338(2q35), CSF1PO (5q33.1), Penta D (21q22.3), TH01(11p15.5), vWA(12p13.31), D21S11(21q21.1), D7S820(7q21.11), D5S818 (5q23.2), TPOX(2p25.3), D8S1179 (8q24.13), D12S391(12p12), D19S433(19q12), and fibrinogen Aα (FGA)(4q28) were genotyped and analyzed as described in our previous study (C. Yang et al., 2017, 2018).

### 2.3 Statistical analysis

Modified‐Powerstates software (Promega) was used to obtain allele and genotype frequencies of the 20 STRs. Statistical analyses were performed using SPSS software version 19.0 as described in our previous study (C. Yang et al., 2017, 2018).

## 3 RESULTS

### 3.1 Hardy–Weinberg equilibrium test

As seen in Table 1, the chi‐squared test was performed on the observed and theoretical genotype frequencies of 20 STR loci in schizophrenia patients and the controls, and the genotype frequency distribution was in line with Hardy–Weinberg equilibrium (*p* > .05).

**TABLE 1:** Hardy–Weinberg equilibrium test for 20 STR loci in male patients and controls

Loci	Group	χ^2^ *p*‐value	Loci	Group	χ^2^ *p*‐value
D3S1368	Patients	χ^2^ = 0.52, *p* = .471	TH01	Patients	χ^2^ = 0.15, *p* = .702
	Controls	χ^2^ = 0.95, *p* = .330		Controls	χ^2^ = 0.01, *p* = .921
D1S1656	Patients	χ^2^ = 0.12, *p* = .729	vWA	Patients	χ^2^ = 0.10, *p* = .753
	Controls	χ^2^ = 0.23, *p* = .633		Controls	χ^2^ = 0.03, *p* = .865
D6S1043	Patients	χ^2^ = 1.87, *p* = .171	D21S11	Patients	χ^2^ = 0.38, *p* = .536
	Controls	χ^2^ = 1.99, *p* = .158		Controls	χ^2^ = 0.06, *p* = .807
D13S317	Patients	χ^2^ = 1.05, *p* = .224	D7S820	Patients	χ^2^ = 0.03, *p* = .857
	Controls	χ^2^ = 0.39, *p* = .531		Controls	χ^2^ = 1.05, *p* = .305
Penta E	Patients	χ^2^ = 0.23, *p* = .633	D5S818	Patients	χ^2^ = 0.02, *p* = .885
	Controls	χ^2^ = 1.37, *p* = .242		Controls	χ^2^ = 1.38, *p* = .241
D16S639	Patients	χ^2^ = 0.13, *p* = .717	TPOX	Patients	χ^2^ = 0.00, *p* = .975
	Controls	χ^2^ = 0.46, *p* = .498		Controls	χ^2^ = 1.72, *p* = .190
D18S51	Patients	χ^2^ = 1.31, *p* = .252	D8S1179	Patients	χ^2^ = 1.72, *p* = .190
	Controls	χ^2^ = 0.02, *p* = .957		Controls	χ^2^ = 0.79, *p* = .373
D2S1338	Patients	χ^2^ = 1.47, *p* = .225	D12S391	Patients	χ^2^ = 1.87, *p* = .174
	Controls	χ^2^ = 0.07, *p* = .792		Controls	χ^2^ = 2.16, *p* = .141
CSF1PO	Patients	χ^2^ = 1.07, *p* = .300	D19S433	Patients	χ^2^ = 0.49, *p* = .484
	Controls	χ^2^ = 0.26, *p* = .213		Controls	χ^2^ = 0.01, *p* = .922
Penta D	Patients	χ^2^ = 0.47, *p* = .494	FGA	Patients	χ^2^ = 0.61, *p* = .434
	Controls	χ^2^ = 0.34, *p* = .559		Controls	χ^2^ = 1.57, *p* = .210

### 3.2 Two STRs showed a difference in the distribution of allele frequency

As seen in Table 2, a remarkable difference existed in the distribution frequency of two alleles (11 and 13) of STR D13S317 between patients and controls. Additionally, in Table 3, a statistically significant difference was observed in the distribution frequency of allele 7 of STR D5S818 between patients and controls. Additionally, there was no obvious difference in the allele distribution frequencies of the other 20 STRs between the two groups (see Supplementary Information).

**TABLE 2:** Comparisons of allele frequencies of the D13S317 locus in male patients (*n* = 355) and controls (*n* = 473; frequency, %)

Alleles	Patients	Controls	χ^2^	*p*‐value	Odds ratio	95%CI
8	197 (27.25)	277 (29.28)	0.47	.4941	0.93	0.75–1.15
9	88 (12.39)	122 (12.90)	0.09	.7612	0.96	0.71–1.28
10	99 (13.94)	130 (13.74)	0.01	.9064	1.02	0.77–1.35
11	206 (29.01)	217 (22.94)	7.87	.0050	1.37	1.10–1.71
12	96 (13.52)	142 (15.01)	0.73	.3925	0.89	0.67–1.17
13	18 (2.4)	44 (4.65)	5.03	.0248	0.53	0.31–0.93
*χ* ^2^	11.74					
*p‐*value	.039					

*Note*: The numbers in brackets indicate frequencies. Allele frequencies less than 1% in both groups were removed. The level of statistical significance for these pairwise tests was set at 0.05/6.

**TABLE 3:** Comparisons of allele frequencies of the D5S818 locus in male patients (*n* = 355) and controls (*n* = 473; frequency, %)

Alleles	Patients	Controls	χ^2^	*p*‐value	Odds ratio	95%CI
7	25 (3.52)	10 (1.06)	11.90	.0006	3.42	1.63–7.16
9	58 (8.17)	77 (8.14)	0.00	.9827	1.00	0.70–1.43
0	137 (19.30)	178 (18.82)	0.06	.8056	1.03	0.81–1.32
11	218 (30.70)	299 (31.61)	0.15	.6949	0.96	0.78–1.18
12	175 (24.65)	231 (24.42)	0.01	.9145	1.01	0.81–1.27
13	90 (12.68)	140 (14.80)	1.53	.2163	0.84	0.63–1.11
*χ* ^2^	13.13					
*p‐*value	.022					

*Note*: The numbers in brackets indicate frequencies. Allele frequencies less than 1% in both groups were removed. The level of statistical significance for these pairwise tests was set at 0.05/6.

### 3.3 Differences in the distribution frequency of genotype of two STRs, D13S317 and D5S818

Then, we examined the genotype at the D13S317 and D5S818 loci. As shown in Tables 4 and 5, univariate analysis further showed that the frequency distribution differences of allele 11 and genotype 10‐11 at the D13S317 locus between the two groups were significant. The frequency distribution differences of allele 7 and genotype 7‐10 at the D5S818 locus between the two groups were significant. No significant differences were observed in the genotype frequency of the remaining 18 STRs between patients and controls (see Supplementary Information).

**TABLE 4:** Comparisons of genotype frequencies of the D13S317 locus in male patients (*n* = 355) and controls (*n* = 473; frequency, %)

Genotype	Patients	Controls	χ^2^	*p*‐value	Odds ratio	95%CI
8‐8	24 (6.76)	37 (7.82)	0.34	.5627	0.85	0.50–1.46
8‐9	28 (7.89)	36 (7.61)	0.02	.8829	1.04	0.62–1.74
8‐10	24 (6.76)	45 (9.51)	2.01	.1560	0.69	0.41–1.16
8‐11	51 (14.37)	68 (14.38)	0.00	.9967	1.00	0.68–1.48
8‐12	38 (10.70)	39 (8.25)	1.45	.2279	1.33	0.83–2.13
8‐13	7 (1.97)	12 (2.54)	0.29	.5909	0.77	0.30–1.98
9‐9	4 (1.13)	4 (0.85)	0.17	.6824	1.34	0.33–5.38
9‐10	9 (2.54)	20 (4.23)	1.72	.1897	0.59	0.27–1.31
9‐11	25 (7.04)	30 (6.34)	0.16	.6890	1.12	0.65–1.94
9‐12	14 (3.94)	20 (4.23)	0.04	.8381	0.93	0.46–1.87
9‐13	2 (0.56)	5 (1.06)	0.59	.4425	0.53	0.10–2.75
10‐10	4 (1.13)	10 (2.11)	1.19	.2754	0.53	0.16–1.70
10‐11	50 (14.08)	19 (4.02)	26.91	.0000002	3.92	2.27–6.77
10‐12	6 (1.69)	18 (3.81)	3.22	.0726	0.44	0.17–1.11
11‐11	22 (6.20)	32 (6.77)	0.11	.7432	0.91	0.52–1.60
11‐12	29 (8.17)	25 (5.29)	2.77	.0963	1.59	0.92–2.77
11‐13	6 (1.69)	11 (2.33)	0.41	.5234	0.72	0.26–1.97
12‐12	3 (0.85)	15 (3.17)	5.16	.0231	0.26	0.08–0.91
12‐13	2 (0.56)	7 (1.48)	1.58	.2081	0.38	0.08–1.83
*χ* ^2^	47.79					
*p‐*value	.0004					

*Note*: The numbers in brackets indicate frequencies. Genotype frequencies less than 1% in both groups were removed. The level of statistical significance for these pairwise tests was set at 0.05/19.

**TABLE 5:** Comparisons of genotype frequencies of the D5S818 locus in male patients (*n* = 355) and controls (*n* = 473; frequency, %)

Genotype	Subject	Controls	χ^2^	*p*‐value	Odds ratio	95%CI
7‐10	12 (3.38)	2 (0.42)	10.67	.0011	8.24	1.83–37.05
7‐11	3 (0.85)	6 (1.27)	0.34	.5609	0.66	0.17–2.67
7‐12	5 (1.41)	2 (0.42)	2.35	.1253	3.36	0.65–17.44
9‐10	8 (2.25)	12 (2.54)	0.07	.7926	0.89	0.36–2.19
9‐11	14 (3.94)	24 (5.07)	0.59	.4418	0.77	0.39–1.51
9‐12	21 (5.92)	24 (5.07)	0.28	.5971	1.18	0.64–2.15
9‐13	6 (1.69)	10 (2.11)	0.19	.6609	0.80	0.29–2.21
10‐10	19 (5.35)	18 (3.81)	1.14	.2864	1.43	0.74–2.77
10‐11	40 (11.27)	52 (10.99)	0.02	.9012	1.03	0.66–1.59
10‐12	27 (7.61)	42 (8.88)	0.43	.5116	0.85	0.51–1.40
10‐13	11 (3.10)	33 (6.98)	6.06	.0138	0.43	0.21–0.86
11‐11	32 (9.01)	48 (10.15)	0.30	.5847	0.88	0.55–1.40
11‐12	63 (17.75)	76 (16.07)	0.41	.5224	1.13	0.78–1.63
11‐13	30 (8.45)	41 (8.67)	0.01	.9120	0.97	0.59–1.59
11‐14	4 (1.13)	1 (0.21)	2.83	.0925	5.38	0.60–48.33
12‐12	15 (4.23)	32 (6.77)	2.44	.1180	0.61	0.32–1.14
12‐13	28 (7.89)	22 (4.65)	3.74	.0530	1.76	0.99–3.12
13‐13	6 (1.69)	16 (3.38)	2.25	.1340	0.49	0.19–1.27
χ^2^	32.94					
*p‐*value	.011					

*Note*: The numbers in brackets indicate frequencies. Genotype frequencies less than 1% in both groups were removed. The level of statistical significance for these pairwise tests was set at 0.05/18.

## 4 DISCUSSION

In this study, by comparing the genetic polymorphisms of 20 STR loci on autosomal chromosome between male schizophrenia patients and male controls, we found a difference in the distribution frequency of some alleles and genotypes of two STRs, indicating that polymorphisms of the D13S317 and D5S818 loci may be associated with male schizophrenia. Specifically, allele 11 and genotype 10‐11 of the D13S317 locus and allele 7 and genotype 7‐10 of the D5S818 locus may be predisposed to male schizophrenia. No correlation was observed in the other autosomal 18 STR loci between the two groups. Nevertheless, these findings should be determined in future investigations.

Our results were consistent with previous studies that suggest STR play a major role in schizophrenia (Alizadeh et al., 2018). Khademi et al. suggested that STR genotypes may be risk factors for schizophrenia (Khademi et al., 2017). The dysregulation of the neurotransmission of glutamate and gamma‐aminobutyric acid (GABA) plays an important role; alterations in a wide range of microcircuits may cause abnormal neurons to manifest in cognitive behavior and social dysfunction (Howes et al., 2015; A. C. Yang & Tsai, 2017). Adequate D2 receptor blockade, intensity and duration of action, and distribution of dopamine can promote its regulation. The regulation of serotonin is associated with a beneficial increase in dopamine release in the striatum. Therefore, it is necessary to determine whether an association exists between STRs and male schizophrenia (Horacek et al., 2006).

Previous research suggested that epigenetic regulation, such as DNA methylation, which increases the risk of schizophrenia, can be used as a potential factor to explain symptom severity and familial genetic variation (Rivollier et al., 2014). Schizophrenia, accounting for about 1% of the world's population, is a complex mental disorder. The genetic pattern of schizophrenia suggests that the risk of non‐Mendelian transmission of psychosis may be attributable to different genetic loci. STR is composed of tandem repeating DNA units scattered throughout the genome with highly heterozygous and evolutionary information (Luan et al., 2020). Additionally, there are many genes located on chromosomes that correlate with schizophrenia, for example, candidate genes of the dopamine system, catechol‐omethyltransferase gene in the human 22q11.2 area (Vevera et al., 2009), monoamine oxidase gene in the Xp11‐23∼11‐4 area (Manuck et al., 2000), and estrogen receptor α gene (Westberg et al., 2003). Taken together, these results suggest a profound genetic basis for schizophrenia. Thus, it is necessary to explore many more candidate genes for male schizophrenia.

In conclusion, this is the first‐time report to suggest that the D13S317 and D5S818 loci are associated with male schizophrenia. Due to the small sample size in this study, its conclusions need to be confirmed by further studies. Meanwhile, only male schizophrenia patients were associated with STR loci in this study; thus, whether there would be any association between female schizophrenia and related STR loci remains to be further studied. The next step of the study was based on the increase in research samples, carrying out high‐density genetic markers fine positioning of the related chromosomal regions, screening the susceptibility genes of schizophrenia, and conducting in‐depth research on how the related genes are related to the occurrence of schizophrenia.

## CONFLICT OF INTEREST

The authors declare no competing interests.

## AUTHOR CONTRIBUTIONS

Xianju Zhou and Huajie Ba designed and conceived the work; Chun Yang, Huajie Ba, and Huihui Zou performed this study and collected the data; Chun Yang, Huajie Ba, Huihui Zou, and Xianju Zhou drafted the article. Chun Yang performed disease diagnosis and collected samples; all authors reviewed the article and approved the final version for publication.

### PEER REVIEW

The peer review history for this article is available at https://publons.com/publon/10.1002/brb3.2637

## Supporting information



Supporting Information

## Data Availability

The data that supports the findings of this study is available from the corresponding author upon reasonable request.
