# Supplementary material for: The association of 20 short tandem repeat loci of autosomal chromosome with male schizophrenia
Source: Brain Behav. 2022 Jun 8;12(7):e2637. doi: 10.1002/brb3.2637 (PMC9304842; doi:10.1002/brb3.2637)
Supplement: Supplementary file 1 — Supporting Information [file BRB3-12-e2637-s001.docx]

Supplementary Information

**The association of 20 short tandem repeat loci on autosomal chromosomes with male schizophrenia.**

Chun Yang^1,*^ , Huajie Ba^2,*^ , HuiHui Zou^3,*^, Xianju Zhou^3^

Supplementary Tables:

Table S1. Comparisons of allele frequencies of D3S1358 locus in male patients and controls

| Alleles | patients（n=355） | controls（n=473） |
| --- | --- | --- |
| 14 | 37 | 39 |
| 15 | 245 | 354 |
| 16 | 214 | 316 |
| 17 | 160 | 185 |
| 18 | 44 | 45 |
| χ2 | 6.63 |  |
| *p-value* | 0.157 |  |

*Note:* *Allele frequency less than 1% in both groups were removed.*

Table S1-1. Comparisons of genotype frequencies of D3S1358 locus in male patients and controls

| Genotype | patients（n=355） | controls（n=473） |
| --- | --- | --- |
| 14-15 | 12 | 16 |
| 14-16 | 12 | 12 |
| 14-17 | 5 | 8 |
| 15-15 | 39 | 69 |
| 15-16 | 73 | 107 |
| 15-17 | 67 | 69 |
| 15-18 | 14 | 20 |
| 16-16 | 32 | 59 |
| 16-17 | 51 | 64 |
| 16-18 | 14 | 13 |
| 17-17 | 13 | 18 |
| 17-18 | 11 | 7 |
| χ2 | 10.98 |  |
| *p-value* | 0.157 |  |

*Note:* *Genotype frequency less than 1% in both groups were removed.*

Table S2. Comparisons of allele frequencies of D1S1656 locus in male patients and controls

| Alleles | patients（n=355） | controls（n=473） |
| --- | --- | --- |
| 11 | 39 | 54 |
| 12 | 39 | 43 |
| 13 | 67 | 93 |
| 14 | 63 | 77 |
| 15 | 201 | 306 |
| 16 | 167 | 230 |
| 17 | 73 | 64 |
| 17.3 | 35 | 48 |
| 18 | 8 | 4 |
| 18.3 | 12 | 22 |
| χ2 | 13.00 |  |
| *p-value* | 0.162 |  |

*Note: Allele frequency less than 1% in both groups were removed.*

Table S2-1. Comparisons of genotype frequencies of D1S1656 locus in male patients and controls

| Genotype | patients（n=355） | controls（n=473） |
| --- | --- | --- |
| 11-13 | 1 | 5 |
| 11-15 | 10 | 14 |
| 11-16 | 12 | 15 |
| 11-17 | 1 | 6 |
| 11-17.3 | 4 | 2 |
| 12-13 | 3 | 7 |
| 12-14 | 5 | 6 |
| 12-15 | 13 | 12 |
| 12-16 | 4 | 9 |
| 12-17 | 5 | 5 |
| 13-13 | 4 | 4 |
| 13-14 | 6 | 6 |
| 13-15 | 24 | 28 |
| 13-16 | 16 | 21 |
| 13-17 | 5 | 5 |
| 13-17.3 | 1 | 8 |
| 14-15 | 10 | 27 |
| 14-16 | 18 | 16 |
| 14-17 | 11 | 4 |
| 15-15 | 28 | 52 |
| 15-16 | 48 | 71 |
| 15-17 | 25 | 25 |
| 15-17.3 | 10 | 16 |
| 15-18.3 | 3 | 7 |
| 16-16 | 21 | 31 |
| 16-17 | 11 | 13 |
| 16-17.3 | 9 | 13 |
| 16-18.3 | 3 | 8 |
| 17-17 | 5 | 1 |
| 17-17.3 | 4 | 3 |
| χ2 | 33.60 |  |
| *p-value* | 0.254 |  |

*Note: Genotype frequency less than 1% in both groups were removed.*

Table S3. Comparisons of allele frequencies of D6S1043 locus in male patients and controls

| Alleles | patients（n=355） | controls（n=473） |
| --- | --- | --- |
| 9 | 7 | 5 |
| 10 | 26 | 33 |
| 11 | 69 | 96 |
| 12 | 108 | 134 |
| 13 | 96 | 127 |
| 14 | 104 | 144 |
| 15 | 9 | 9 |
| 16 | 4 | 1 |
| 17 | 27 | 39 |
| 18 | 116 | 168 |
| 19 | 100 | 146 |
| 20 | 30 | 34 |
| 21 | 9 | 6 |
| χ2 | 8.16 |  |
| *p-value* | 0.773 |  |

*Note:* *Allele frequency less than 1% in both groups were removed.*

Table S3-1. Comparisons of genotype frequencies of D6S1043 locus in male patients and controls

| Genotype | patients（n=355） | controls（n=473） |
| --- | --- | --- |
| 10-11 | 4 | 3 |
| 10-12 | 3 | 7 |
| 10-14 | 4 | 5 |
| 10-18 | 3 | 8 |
| 10-19 | 5 | 5 |
| 11-11 | 0 | 5 |
| 11-12 | 9 | 16 |
| 11-13 | 9 | 14 |
| 11-14 | 14 | 10 |
| 11-17 | 8 | 1 |
| 11-18 | 8 | 20 |
| 11-19 | 10 | 15 |
| 11-20 | 6 | 4 |
| 12-12 | 14 | 11 |
| 12-13 | 14 | 17 |
| 12-14 | 10 | 22 |
| 12-17 | 2 | 6 |
| 12-18 | 17 | 17 |
| 12-19 | 17 | 21 |
| 13-13 | 9 | 7 |
| 13-14 | 9 | 18 |
| 13-18 | 16 | 26 |
| 13-19 | 18 | 17 |
| 13-20 | 3 | 9 |
| 14-14 | 9 | 13 |
| 14-17 | 4 | 9 |
| 14-18 | 16 | 25 |
| 14-19 | 19 | 23 |
| 14-20 | 7 | 2 |
| 17-18 | 5 | 5 |
| 18-18 | 15 | 17 |
| 18-19 | 11 | 25 |
| 18-20 | 4 | 5 |
| 19-19 | 5 | 15 |
| χ2 | 42.16 |  |
| *p-value* | 0.132 |  |

*Note: Genotype frequency less than 1% in both groups were removed.*

Table S4. Comparisons of allele frequencies of D13S317 locus in male patients and controls

| Alleles | patients（n=355） | controls（n=473） | χ2 | p-value | OR | 95%CI |
| --- | --- | --- | --- | --- | --- | --- |
| 8 | 197（27.25） | 277（29.28） | 0.47 | 0.4941 | 0.93 | 0.75-1.15 |
| 9 | 88（12.39） | 122（12.90） | 0.09 | 0.7612 | 0.96 | 0.71-1.28 |
| 10 | 99（13.94） | 130（13.74） | 0.01 | 0.9064 | 1.02 | 0.77-1.35 |
| 11 | 206（29.01） | 217（22.94） | 7.87 | 0.0050 | 1.37 | 1.10-1.71 |
| 12 | 96（13.52） | 142（15.01） | 0.73 | 0.3925 | 0.89 | 0.67-1.17 |
| 13 | 18（2.4） | 44（4.65） | 5.03 | 0.0248 | 0.53 | 0.31-0.93 |

*Note: The numbers in brackets indicate frequency (%); Allele frequency less than 1% in both groups were removed*

Table S4-1. Comparisons of genotype frequencies of D13S317 locus in male patients and controls

| Genotype | patients（n=355） | controls（n=473） | χ2 | p-value | OR | 95%CI |
| --- | --- | --- | --- | --- | --- | --- |
| 8-8 | 24（6.76） | 37（7.82） | 0.34 | 0.5627 | 0.85 | 0.50-1.46 |
| 8-9 | 28（7.89） | 36（7.61） | 0.02 | 0.8829 | 1.04 | 0.62-1.74 |
| 8-10 | 24（6.76） | 45（9.51） | 2.01 | 0.1560 | 0.69 | 0.41-1.16 |
| 8-11 | 51（14.37） | 68（14.38） | 0.00 | 0.9967 | 1.00 | 0.68-1.48 |
| 8-12 | 38（10.70） | 39（8.25） | 1.45 | 0.2279 | 1.33 | 0.83-2.13 |
| 8-13 | 7（1.97） | 12（2.54） | 0.29 | 0.5909 | 0.77 | 0.30-1.98 |
| 9-9 | 4（1.13） | 4（0.85） | 0.17 | 0.6824 | 1.34 | 0.33-5.38 |
| 9-10 | 9（2.54） | 20（4.23） | 1.72 | 0.1897 | 0.59 | 0.27-1.31 |
| 9-11 | 25（7.04） | 30（6.34） | 0.16 | 0.6890 | 1.12 | 0.65-1.94 |
| 9-12 | 14（3.94） | 20（4.23） | 0.04 | 0.8381 | 0.93 | 0.46-1.87 |
| 9-13 | 2（0.56） | 5（1.06） | 0.59 | 0.4425 | 0.53 | 0.10-2.75 |
| 10-10 | 4（1.13） | 10（2.11） | 1.19 | 0.2754 | 0.53 | 0.16-1.70 |
| 10-11 | 50（14.08） | 19（4.02） | 26.91 | 0.0000002 | 3.92 | 2.27-6.77 |
| 10-12 | 6（1.69） | 18（3.81） | 3.22 | 0.0726 | 0.44 | 0.17-1.11 |
| 11-11 | 22（6.20） | 32（6.77） | 0.11 | 0.7432 | 0.91 | 0.52-1.60 |
| 11-12 | 29（8.17） | 25（5.29） | 2.77 | 0.0963 | 1.59 | 0.92-2.77 |
| 11-13 | 6（1.69） | 11（2.33） | 0.41 | 0.5234 | 0.72 | 0.26-1.97 |
| 12-12 | 3（0.85） | 15（3.17） | 5.16 | 0.0231 | 0.26 | 0.08-0.91 |
| 12-13 | 2（0.56） | 7（1.48） | 1.58 | 0.2081 | 0.38 | 0.08-1.83 |

*Note: The numbers in brackets indicate frequency (%); Genotype frequency less than 1% in both groups were removed.*

Table S5. Comparisons of allele frequencies of Penta E locus in male patients and controls

| Alleles | patients （n=355） | controls（n=473） |
| --- | --- | --- |
| 5 | 30 | 56 |
| 10 | 38 | 49 |
| 11 | 98 | 117 |
| 12 | 72 | 109 |
| 13 | 41 | 52 |
| 14 | 55 | 62 |
| 15 | 69 | 103 |
| 16 | 49 | 92 |
| 17 | 59 | 81 |
| 18 | 47 | 65 |
| 19 | 53 | 53 |
| 20 | 33 | 36 |
| 21 | 25 | 30 |
| 22 | 15 | 16 |
| χ2 | 12.29 |  |
| *p-value* | 0.504 |  |

*Note: Allele frequency less than 1% in both groups were removed*

Table S5-1. Comparisons of genotype frequencies of Penta E locus in male patients and controls

| Genotype | patients（n=355） | controls（4n=73） |
| --- | --- | --- |
| 5-11 | 7 | 4 |
| 5-12 | 3 | 8 |
| 5-15 | 0 | 7 |
| 5-17 | 3 | 6 |
| 5-18 | 3 | 6 |
| 10-11 | 2 | 9 |
| 10-12 | 4 | 4 |
| 10-15 | 4 | 5 |
| 10-16 | 2 | 6 |
| 10-17 | 6 | 5 |
| 11-11 | 7 | 7 |
| 11-12 | 7 | 10 |
| 11-13 | 8 | 11 |
| 11-14 | 10 | 7 |
| 11-15 | 12 | 19 |
| 11-16 | 3 | 8 |
| 11-17 | 8 | 6 |
| 11-18 | 5 | 7 |
| 11-19 | 7 | 9 |
| 11-20 | 7 | 7 |
| 11-21 | 4 | 3 |
| 12-12 | 4 | 6 |
| 12-13 | 5 | 4 |
| 12-14 | 6 | 6 |
| 12-15 | 9 | 11 |
| 12-16 | 3 | 12 |
| 12-17 | 2 | 9 |
| 12-18 | 8 | 12 |
| 12-19 | 5 | 8 |
| 12-20 | 4 | 4 |
| 12-21 | 4 | 3 |
| 13-15 | 2 | 7 |
| 13-17 | 4 | 6 |
| 13-18 | 4 | 3 |
| 13-19 | 4 | 5 |
| 13-20 | 5 | 0 |
| 14-14 | 1 | 5 |
| 14-15 | 4 | 3 |
| 14-16 | 5 | 3 |
| 14-17 | 7 | 2 |
| 14-18 | 3 | 8 |
| 14-19 | 5 | 3 |
| 14-20 | 4 | 3 |
| 15-15 | 4 | 6 |
| 15-16 | 4 | 9 |
| 15-17 | 9 | 5 |
| 15-18 | 7 | 6 |
| 15-19 | 7 | 1 |
| 15-20 | 0 | 5 |
| 15-21 | 2 | 5 |
| 16-16 | 1 | 7 |
| 16-17 | 8 | 11 |
| 16-18 | 6 | 8 |
| 16-19 | 6 | 3 |
| 16-20 | 1 | 9 |
| 16-21 | 1 | 6 |
| 17-18 | 1 | 5 |
| 17-19 | 4 | 8 |
| χ2 | 75.60 |  |
| *p-value* | 0.050 |  |

*Note: Genotype frequency less than 1% in both groups were removed.*

Table S6. Comparisons of allele frequencies of D16S639 locus in male patients and controls

| Alleles | patients（n=355） | controls（n=473） |
| --- | --- | --- |
| 8 | 3 | 10 |
| 9 | 205 | 269 |
| 10 | 100 | 111 |
| 11 | 168 | 240 |
| 12 | 153 | 217 |
| 13 | 71 | 88 |
| 14 | 7 | 10 |

*Note: Allele frequency less than 1% in both groups were removed*

Table S6-1. Comparisons of genotype frequencies of D16S639 locus in male patients and controls

| Genotype | patients（n=355） | controls（n=473） |
| --- | --- | --- |
| 8-11 | 1 | 5 |
| 9-9 | 32 | 45 |
| 9-10 | 27 | 32 |
| 9-11 | 52 | 63 |
| 9-12 | 38 | 54 |
| 9-13 | 19 | 25 |
| 10-10 | 5 | 8 |
| 10-11 | 29 | 29 |
| 10-12 | 23 | 20 |
| 10-13 | 10 | 11 |
| 11-11 | 21 | 25 |
| 11-12 | 34 | 71 |
| 11-13 | 8 | 19 |
| 12-12 | 16 | 25 |
| 12-13 | 23 | 19 |
| 13-13 | 5 | 7 |

*Note: Genotype frequency less than 1% in both groups were removed.*

Table S7. Comparisons of allele frequencies of D18S51 locus in male patients and controls

| Alleles | patients（n=355） | controls（n=473） |
| --- | --- | --- |
| 12 | 20 | 28 |
| 13 | 145 | 198 |
| 14 | 146 | 204 |
| 15 | 126 | 165 |
| 16 | 91 | 116 |
| 17 | 59 | 69 |
| 18 | 34 | 38 |
| 19 | 37 | 47 |
| 20 | 17 | 21 |
| 21 | 19 | 23 |
| 22 | 7 | 20 |
| χ2 | 4.84 |  |
| *p-value* | 0.906 |  |

*Note: Allele frequency less than 1% in both groups were removed*

Table S7-1. Comparisons of genotype frequencies of D18S51 locus in male patients and controls

| Genotype | patients（n=355） | controls（n=473） |
| --- | --- | --- |
| 12-13 | 6 | 6 |
| 12-14 | 4 | 4 |
| 12-15 | 4 | 6 |
| 13-13 | 19 | 17 |
| 13-14 | 24 | 44 |
| 13-15 | 24 | 33 |
| 13-16 | 19 | 22 |
| 13-17 | 8 | 19 |
| 13-18 | 9 | 7 |
| 13-19 | 5 | 12 |
| 13-20 | 5 | 5 |
| 13-21 | 1 | 5 |
| 13-22 | 4 | 8 |
| 14-14 | 17 | 22 |
| 14-15 | 21 | 35 |
| 14-16 | 26 | 22 |
| 14-17 | 11 | 20 |
| 14-18 | 8 | 8 |
| 14-19 | 7 | 7 |
| 14-20 | 3 | 6 |
| 14-21 | 6 | 6 |
| 15-15 | 9 | 15 |
| 15-16 | 19 | 22 |
| 15-17 | 15 | 7 |
| 15-18 | 3 | 9 |
| 15-19 | 13 | 7 |
| 15-21 | 4 | 4 |
| 15-22 | 0 | 5 |
| 16-16 | 7 | 11 |
| 16-17 | 5 | 2 |
| 16-18 | 1 | 7 |
| 16-19 | 2 | 9 |
| 17-17 | 4 | 2 |
| 17-19 | 3 | 5 |
| χ2 | 12.25 |  |
| *p-value* | 0.234 |  |

*Note: Genotype frequency less than 1% in both groups were removed.*

Table S8. Comparisons of allele frequencies of D2S1338 locus in male patients and controls

| Alleles | patients（n=355） | controls（n=473） |
| --- | --- | --- |
| 16 | 12 | 9 |
| 17 | 43 | 59 |
| 18 | 87 | 120 |
| 19 | 120 | 155 |
| 20 | 87 | 117 |
| 21 | 17 | 27 |
| 22 | 37 | 47 |
| 23 | 147 | 190 |
| 24 | 109 | 158 |
| 25 | 42 | 46 |
| 26 | 7 | 13 |
| χ2 | 4.15 |  |
| *p-value* | 0.940 |  |

*Note: Allele frequency less than 1% in both groups were removed*

Table S8-1. Comparisons of genotype frequencies of D2S1338 locus in male patients and controls

| Genotype | patients（n=355） | controls（n=473） |
| --- | --- | --- |
| 16-23 | 2 | 5 |
| 17-18 | 4 | 5 |
| 17-19 | 12 | 11 |
| 17-20 | 6 | 8 |
| 17-21 | 1 | 7 |
| 17-23 | 7 | 12 |
| 17-24 | 6 | 6 |
| 18-18 | 4 | 7 |
| 18-19 | 14 | 21 |
| 18-20 | 9 | 16 |
| 18-22 | 6 | 11 |
| 18-23 | 14 | 20 |
| 18-24 | 19 | 22 |
| 18-25 | 9 | 4 |
| 19-19 | 8 | 12 |
| 19-20 | 13 | 14 |
| 19-22 | 7 | 8 |
| 19-23 | 22 | 41 |
| 19-24 | 22 | 25 |
| 19-25 | 9 | 6 |
| 20-20 | 3 | 8 |
| 20-22 | 2 | 7 |
| 20-23 | 26 | 23 |
| 20-24 | 15 | 26 |
| 20-25 | 5 | 4 |
| 21-24 | 3 | 7 |
| 22-23 | 8 | 7 |
| 23-23 | 17 | 17 |
| 23-24 | 25 | 25 |
| 23-25 | 8 | 15 |
| 24-24 | 4 | 16 |
| 24-25 | 6 | 7 |
| χ2 | 28.29 |  |
| *p-value* | 0.606 |  |

*Note: Genotype frequency less than 1% in both groups were removed.*

Table S9. Comparisons of allele frequencies of CSF1PO locus in male patients and controls

| Alleles | patients（n=355） | controls（n=473） |
| --- | --- | --- |
| 9 | 35 | 31 |
| 10 | 165 | 228 |
| 11 | 189 | 231 |
| 12 | 264 | 382 |
| 13 | 44 | 61 |
| 14 | 11 | 9 |
| χ2 | 5.98 |  |
| *p-value* | 0.308 |  |

*Note*: *Allele frequency less than 1% in both groups were removed*

Table S9-1. Comparisons of genotype frequencies of CSF1PO locus in male patients and controls

| Genotype | patients（n=355） | controls（n=473） |
| --- | --- | --- |
| 9-10 | 10 | 6 |
| 9-11 | 5 | 7 |
| 9-12 | 11 | 14 |
| 10-10 | 19 | 27 |
| 10-11 | 44 | 58 |
| 10-12 | 58 | 91 |
| 10-13 | 11 | 14 |
| 11-11 | 27 | 26 |
| 11-12 | 70 | 97 |
| 11-13 | 13 | 14 |
| 12-12 | 54 | 75 |
| 12-13 | 13 | 26 |
| χ2 | 6.78 |  |
| *p-value* | 0.817 |  |

*Note: Genotype frequency less than 1% in both groups were removed.*

Table S10. Comparisons of allele frequencies of Penta D locus in male patients and controls

| Alleles | patients（n=355） | controls（n=473） |
| --- | --- | --- |
| 8 | 38 | 52 |
| 9 | 218 | 305 |
| 10 | 74 | 108 |
| 11 | 110 | 135 |
| 12 | 134 | 182 |
| 13 | 94 | 113 |
| 14 | 30 | 37 |
| χ2 | 1.76 |  |
| *p-value* | 0.940 |  |

*Note: Allele frequency less than 1% in both groups were removed*

Table S10-1. Comparisons of genotype frequencies of Penta D locus in male patients and controls

| Genotype | patients（n=355） | controls（n=473） |
| --- | --- | --- |
| 8-9 | 13 | 17 |
| 8-10 | 4 | 4 |
| 8-11 | 4 | 6 |
| 8-12 | 11 | 15 |
| 8-13 | 5 | 6 |
| 9-9 | 29 | 45 |
| 9-10 | 19 | 34 |
| 9-11 | 38 | 48 |
| 9-12 | 43 | 67 |
| 9-13 | 30 | 32 |
| 9-14 | 13 | 11 |
| 10-10 | 6 | 11 |
| 10-11 | 12 | 11 |
| 10-12 | 18 | 20 |
| 10-13 | 8 | 14 |
| 11-11 | 13 | 10 |
| 11-12 | 11 | 23 |
| 11-13 | 10 | 20 |
| 11-14 | 7 | 5 |
| 12-12 | 13 | 13 |
| 12-13 | 18 | 21 |
| 12-14 | 5 | 9 |
| 13-13 | 9 | 6 |
| 13-14 | 2 | 6 |
| χ2 | 15.75 |  |
| *p-value* | 0.866 |  |

*Note*: *Genotype frequency less than 1% in both groups were removed.*

Table S11. Comparisons of allele frequencies of TH01 locus in male patients and controls

| Alleles | patients（n=355） | controls（n=473） |
| --- | --- | --- |
| 6 | 79 | 86 |
| 7 | 180 | 250 |
| 8 | 37 | 45 |
| 9 | 369 | 508 |
| 9.3 | 25 | 29 |
| 10 | 19 | 28 |
| χ2 | 2.64 |  |
| p-value | 0.756 |  |

*Note: Allele frequency less than 1% in both groups were removed*

Table S11-1. Comparisons of genotype frequencies of TH01 locus in male patients and controls

| Genotype | patients（n=355） | controls（n=473） |
| --- | --- | --- |
| 6-6 | 6 | 5 |
| 6-7 | 21 | 30 |
| 6-9 | 38 | 40 |
| 6-9.3 | 6 | 2 |
| 7-7 | 19 | 32 |
| 7-8 | 7 | 7 |
| 7-9 | 104 | 136 |
| 7-9.3 | 8 | 7 |
| 7-10 | 2 | 6 |
| 8-9 | 17 | 28 |
| 9-9 | 93 | 136 |
| 9-9.3 | 8 | 16 |
| 9-10 | 15 | 16 |
| χ2 | 10.50 |  |
| *p-value* | 0.612 |  |

*Note: Genotype frequency less than 1% in both groups were removed.*

Table S12. Comparisons of allele frequencies of VWA locus in male patients and controls

| Alleles | patients（n=355） | controls（n=473） |
| --- | --- | --- |
| 14 | 173 | 231 |
| 15 | 22 | 21 |
| 16 | 127 | 172 |
| 17 | 163 | 244 |
| 18 | 141 | 159 |
| 19 | 66 | 95 |
| 20 | 16 | 22 |
| χ2 | 4.88 |  |
| *p-value* | 0.559 |  |

*Note: Allele frequency less than 1% in both groups were removed*

Table S12-1. Comparisons of genotype frequencies of VWA locus in male patients and controls

| Genotype | patients（n=355） | controls（n=473） |
| --- | --- | --- |
| 14-14 | 21 | 34 |
| 14-15 | 6 | 6 |
| 14-16 | 30 | 40 |
| 14-17 | 36 | 51 |
| 14-18 | 41 | 41 |
| 14-19 | 13 | 20 |
| 14-20 | 5 | 5 |
| 15-17 | 10 | 8 |
| 16-16 | 11 | 15 |
| 16-17 | 29 | 41 |
| 16-18 | 25 | 32 |
| 16-19 | 17 | 19 |
| 16-20 | 2 | 6 |
| 17-17 | 21 | 30 |
| 17-18 | 32 | 45 |
| 17-19 | 11 | 32 |
| 17-20 | 3 | 5 |
| 18-18 | 10 | 12 |
| 18-19 | 15 | 12 |
| 18-20 | 5 | 3 |
| χ2 | 14.03 |  |
| *p-value* | 0.782 |  |

*Note: Genotype frequency less than 1% in both groups were removed.*

Table S13. Comparisons of allele frequencies of D21S11 locus in male patients and controls

| Alleles | patients（n=355） | controls（n=473） |  |  |
| --- | --- | --- | --- | --- |
| 28 | 36 | 34 |  |  |
| 29 | 192 | 230 |  |  |
| 30 | 186 | 300 |  |  |
| 30.2 | 2 | 10 |  |  |
| 30.3 | 9 | 4 |  |  |
| 31 | 85 | 103 |  |  |
| 31.2 | 56 | 76 |  |  |
| 32 | 23 | 33 |  |  |
| 32.2 | 78 | 101 |  |  |
| 33.2 | 29 | 34 |  |  |
| χ2 | 15.32 |  |  |  |
| *p-value* | 0.082 |  |  |  |

*Note: Allele frequency less than 1% in both groups were removed*

Table S13-1. Comparisons of genotype frequencies of D21S11 locus in male patients and controls

| Genotype | patients（n=355） | controls（n=473） |
| --- | --- | --- |
| 28-29 | 11 | 8 |
| 28-30 | 8 | 13 |
| 28-31 | 5 | 4 |
| 28-32.2 | 5 | 3 |
| 29-29 | 25 | 25 |
| 29-30 | 49 | 79 |
| 29-31 | 25 | 22 |
| 29-31.2 | 17 | 23 |
| 29-32 | 5 | 5 |
| 29-32.2 | 17 | 29 |
| 29-33.2 | 10 | 7 |
| 30-30 | 25 | 50 |
| 30-30.2 | 1 | 6 |
| 30-31 | 16 | 36 |
| 30-31.2 | 18 | 17 |
| 30-32 | 9 | 12 |
| 30-32.2 | 26 | 24 |
| 30-33.2 | 4 | 6 |
| 31-31 | 4 | 4 |
| 31-31.2 | 10 | 9 |
| 31-32.2 | 10 | 10 |
| 31-33.2 | 2 | 7 |
| 31.2-32.2 | 5 | 7 |
| 31.2-33.2 | 3 | 6 |
| 32-32.2 | 4 | 8 |
| 32.2-32.2 | 4 | 6 |
| χ2 | 25.35 |  |
| *p-value* | 0.443 |  |

*Note:* *Genotype frequency less than 1% in both groups were removed*

Table S14. Comparisons of allele frequencies of D7S820 locus in male patients and controls

| Alleles | patients（n=355） | controls（n=473） |
| --- | --- | --- |
| 8 | 88 | 135 |
| 9 | 48 | 50 |
| 10 | 132 | 158 |
| 11 | 239 | 328 |
| 12 | 172 | 226 |
| 13 | 28 | 37 |
| χ2 | 3.49 |  |
| *p-value* | 0.625 |  |

*Note: Allele frequency less than 1% in both groups were removed*

Table S14-1. Comparisons of genotype frequencies of D7S820 locus in male patients and controls

| Genotype | patients（n=355） | controls（n=473） |
| --- | --- | --- |
| 8-8 | 6 | 11 |
| 8-9 | 8 | 6 |
| 8-10 | 12 | 24 |
| 8-11 | 26 | 48 |
| 8-12 | 24 | 31 |
| 8-13 | 5 | 4 |
| 9-10 | 10 | 5 |
| 9-11 | 15 | 19 |
| 9-12 | 11 | 14 |
| 10-10 | 14 | 11 |
| 10-11 | 46 | 54 |
| 10-12 | 28 | 41 |
| 10-13 | 7 | 11 |
| 11-11 | 40 | 54 |
| 11-12 | 62 | 83 |
| 11-13 | 10 | 10 |
| 12-12 | 21 | 22 |
| 12-13 | 4 | 8 |
| χ2 | 12.62 |  |
| *p-value* | 0.721 |  |

*Note: Genotype frequency less than 1% in both groups were removed*

Table S15. Comparisons of allele frequencies of D5S818 locus in male patients and controls

| Alleles | patients（n=355） | controls（n=473） | χ2 | p-value | OR | 95%CI |
| --- | --- | --- | --- | --- | --- | --- |
| 7 | 25（3.52） | 10（1.06） | 11.90 | 0.0006 | 3.42 | 1.63-7.16 |
| 9 | 58（8.17） | 77（8.14） | 0.00 | 0.9827 | 1.00 | 0.70-1.43 |
| 10 | 137（19.30） | 178（18.82） | 0.06 | 0.8056 | 1.03 | 0.81-1.32 |
| 11 | 218（30.70） | 299（31.61） | 0.15 | 0.6949 | 0.96 | 0.78-1.18 |
| 12 | 175（24.65） | 231（24.42） | 0.01 | 0.9145 | 1.01 | 0.81-1.27 |
| 13 | 90（12.68） | 140（14.80） | 1.53 | 0.2163 | 0.84 | 0.63-1.11 |

*Note: The numbers in brackets indicate frequency (%); Allele frequency less than 1% in both groups were removed*

Table S15-1. Comparisons of genotype frequencies of D5S818 locus in male patients and controls

| Genotype | patients（n=355） | controls（n=473） | χ2 | p-value | OR | 95%CI |
| --- | --- | --- | --- | --- | --- | --- |
| 7-10 | 12（3.38） | 2（0.42） | 10.67 | 0.0011 | 8.24 | 1.83-37.05 |
| 7-11 | 3（0.85） | 6（1.27） | 0.34 | 0.5609 | 0.66 | 0.17-2.67 |
| 7-12 | 5（1.41） | 2（0.42） | 2.35 | 0.1253 | 3.36 | 0.65-17.44 |
| 9-10 | 8（2.25） | 12（2.54） | 0.07 | 0.7926 | 0.89 | 0.36-2.19 |
| 9-11 | 14（3.94） | 24（5.07） | 0.59 | 0.4418 | 0.77 | 0.39-1.51 |
| 9-12 | 21（5.92） | 24（5.07） | 0.28 | 0.5971 | 1.18 | 0.64-2.15 |
| 9-13 | 6（1.69） | 10（2.11） | 0.19 | 0.6609 | 0.80 | 0.29-2.21 |
| 10-10 | 19（5.35） | 18（3.81） | 1.14 | 0.2864 | 1.43 | 0.74-2.77 |
| 10-11 | 40（11.27） | 52（10.99） | 0.02 | 0.9012 | 1.03 | 0.66-1.59 |
| 10-12 | 27（7.61） | 42（8.88） | 0.43 | 0.5116 | 0.85 | 0.51-1.40 |
| 10-13 | 11（3.10） | 33（6.98） | 6.06 | 0.0138 | 0.43 | 0.21-0.86 |
| 11-11 | 32（9.01） | 48（10.15） | 0.30 | 0.5847 | 0.88 | 0.55-1.40 |
| 11-12 | 63（17.75） | 76（16.07） | 0.41 | 0.5224 | 1.13 | 0.78-1.63 |
| 11-13 | 30（8.45） | 41（8.67） | 0.01 | 0.9120 | 0.97 | 0.59-1.59 |
| 11-14 | 4（1.13） | 1（0.21） | 2.83 | 0.0925 | 5.38 | 0.60-48.33 |
| 12-12 | 15（4.23） | 32（6.77） | 2.44 | 0.1180 | 0.61 | 0.32-1.14 |
| 12-13 | 28（7.89） | 22（4.65） | 3.74 | 0.0530 | 1.76 | 0.99-3.12 |
| 13-13 | 6（1.69） | 16（3.38） | 2.25 | 0.1340 | 0.49 | 0.19-1.27 |

*Note: The numbers in brackets indicate frequency (%); Genotype frequency less than 1% in both groups were removed*

Table S16. Comparisons of allele frequencies of TPOX locus in male patients and controls

| Alleles | patients（n=355） | controls（n=473） |
| --- | --- | --- |
| 8 | 375 | 467 |
| 9 | 93 | 138 |
| 10 | 19 | 27 |
| 11 | 195 | 292 |
| 12 | 25 | 20 |
| χ2 | 6.19 |  |
| *p-value* | 0.185 |  |

*Note: Allele frequency less than 1% in both groups were removed*

Table S16-1. Comparisons of genotype frequencies of TPOX locus in male patients and controls

| Genotype | patients（n=355） | controls（n=473） |
| --- | --- | --- |
| 8-8 | 100 | 106 |
| 8-9 | 46 | 75 |
| 8-10 | 14 | 19 |
| 8-11 | 102 | 148 |
| 8-12 | 11 | 11 |
| 9-9 | 6 | 8 |
| 9-11 | 30 | 44 |
| 9-12 | 5 | 3 |
| 10-11 | 5 | 8 |
| 11-11 | 25 | 43 |
| 11-12 | 7 | 6 |
| χ2 | 7.97 |  |
| *p-value* | 0.632 |  |

*Note: Genotype frequency less than 1% in both groups were removed*

Table S17. Comparisons of allele frequencies of D8S1179 locus in male patients and controls

| Alleles | patients（n=355） | controls（n=473） |
| --- | --- | --- |
| 10 | 76 | 94 |
| 11 | 62 | 68 |
| 12 | 77 | 127 |
| 13 | 138 | 213 |
| 14 | 144 | 182 |
| 15 | 142 | 162 |
| 16 | 64 | 78 |
| 17 | 6 | 18 |
| χ2 | 10.92 |  |
| *p-value* | 0.142 |  |

*Note: Allele frequency less than 1% in both groups were removed*

Table S17-1. Comparisons of genotype frequencies of D8S1179 locus in male patients and controls

| Genotype | patients（n=355） | controls（n=473） |
| --- | --- | --- |
| 10-11 | 7 | 6 |
| 10-12 | 8 | 15 |
| 10-13 | 21 | 22 |
| 10-14 | 14 | 16 |
| 10-15 | 10 | 15 |
| 10-16 | 9 | 11 |
| 11-11 | 4 | 2 |
| 11-12 | 5 | 10 |
| 11-13 | 12 | 16 |
| 11-14 | 15 | 14 |
| 11-15 | 9 | 14 |
| 11-16 | 5 | 3 |
| 12-12 | 3 | 8 |
| 12-13 | 14 | 29 |
| 12-14 | 14 | 25 |
| 12-15 | 20 | 12 |
| 12-16 | 8 | 18 |
| 13-13 | 6 | 28 |
| 13-14 | 35 | 39 |
| 13-15 | 30 | 39 |
| 13-16 | 12 | 10 |
| 14-14 | 15 | 20 |
| 14-15 | 28 | 28 |
| 14-16 | 7 | 13 |
| 15-15 | 13 | 17 |
| 15-16 | 19 | 15 |
| χ2 | 31.19 |  |
| *p-value* | 0.183 |  |

*Note: Genotype frequency less than 1% in both groups were removed*

Table S18. Comparisons of allele frequencies of D12S391locus in male patients and controls

| Alleles | patients（n=355） | controls（n=473） |
| --- | --- | --- |
| 15 | 10 | 16 |
| 16 | 2 | 8 |
| 17 | 65 | 97 |
| 18 | 160 | 201 |
| 19 | 168 | 194 |
| 20 | 113 | 155 |
| 21 | 75 | 119 |
| 22 | 68 | 74 |
| 23 | 32 | 50 |
| 24 | 6 | 17 |
| 25 | 10 | 13 |
| χ2 | 11.06 |  |
| *p-value* | 0.353 |  |

*Note: Allele frequency less than 1% in both groups were removed*

Table S18-1. Comparisons of genotype frequencies of D12S391 locus in male patients and controls

| Genotype | patients（n=355） | controls（n=473） |
| --- | --- | --- |
| 15-18 | 4 | 3 |
| 17-17 | 1 | 5 |
| 17-18 | 16 | 16 |
| 17-19 | 12 | 25 |
| 17-20 | 13 | 14 |
| 17-21 | 11 | 17 |
| 17-22 | 6 | 6 |
| 18-18 | 19 | 22 |
| 18-19 | 40 | 41 |
| 18-20 | 24 | 27 |
| 18-21 | 13 | 31 |
| 18-22 | 14 | 17 |
| 18-23 | 4 | 14 |
| 18-24 | 1 | 7 |
| 18-25 | 5 | 0 |
| 19-19 | 23 | 22 |
| 19-20 | 26 | 33 |
| 19-21 | 22 | 20 |
| 19-22 | 10 | 13 |
| 19-23 | 10 | 7 |
| 20-20 | 11 | 20 |
| 20-21 | 9 | 15 |
| 20-22 | 9 | 12 |
| 20-23 | 7 | 10 |
| 21-21 | 3 | 8 |
| 21-22 | 6 | 10 |
| 22-22 | 8 | 3 |
| χ2 | 33.69 |  |
| *p-value* | 0.143 |  |

*Note: Genotype frequency less than 1% in both groups were removed*

Table S19. Comparisons of allele frequencies of D19S433 locus in male patients and controls

| Alleles |  | patients（n=355） | controls（n=473） |
| --- | --- | --- | --- |
| 12 |  | 19 | 34 |
| 13 |  | 211 | 271 |
| 13.2 |  | 41 | 33 |
| 14 |  | 175 | 240 |
| 14.2 |  | 64 | 102 |
| 15 |  | 52 | 68 |
| 15.2 |  | 101 | 140 |
| 16 |  | 9 | 9 |
| 16.2 |  | 35 | 38 |
| χ2 |  | 8.53 |  |
| *p-value* |  | 0.383 |  |

*Note: Allele frequency less than 1% in both groups were removed*

Table S19-1. Comparisons of genotype frequencies of D19S433 locus in male patients and controls

| Genotype | patients（n=355） | controls（n=473） |
| --- | --- | --- |
| 12-13 | 4 | 8 |
| 12-14 | 7 | 11 |
| 12-14.2 | 3 | 5 |
| 13-13 | 37 | 34 |
| 13-13.2 | 8 | 9 |
| 13-14 | 50 | 79 |
| 13-14.2 | 27 | 25 |
| 13-15 | 12 | 27 |
| 13-15.2 | 25 | 36 |
| 13-16.2 | 7 | 14 |
| 13.2-14 | 7 | 6 |
| 13.2-15 | 8 | 3 |
| 13.2-15.2 | 9 | 6 |
| 14-14 | 20 | 31 |
| 14-14.2 | 19 | 26 |
| 14-15 | 19 | 11 |
| 14-15.2 | 25 | 29 |
| 14-16.2 | 7 | 10 |
| 14.2-14.2 | 1 | 5 |
| 14.2-15 | 2 | 10 |
| 14.2-15.2 | 5 | 18 |
| 14.2-16.2 | 6 | 4 |
| 15-15.2 | 6 | 8 |
| 15.2-15.2 | 9 | 17 |
| 15.2-16.2 | 7 | 3 |
| χ2 | 35.31 |  |
| *p-value* | 0.064 |  |

*Note: Genotype frequency less than 1% in both groups were removed*

Table S20. Comparisons of allele frequencies of FGA locus in male patients and controls

| Alleles | patients（n=355） | controls（n=473） |
| --- | --- | --- |
| 18 | 19 | 21 |
| 19 | 36 | 46 |
| 20 | 26 | 41 |
| 21 | 82 | 89 |
| 22 | 126 | 170 |
| 22.2 | 4 | 6 |
| 23 | 174 | 242 |
| 23.2 | 10 | 11 |
| 24 | 123 | 144 |
| 24.2 | 1 | 6 |
| 25 | 61 | 102 |
| 26 | 36 | 45 |
| 27 | 4 | 10 |
| 28 | 4 | 2 |
| χ2 | 11.10 |  |
| *p-value* | 0.602 |  |

*Note: Allele frequency less than 1% in both groups were removed*

Table S20-1. Comparisons of genotype frequencies of FGA locus in male patients and controls

| Genotype | patients（n=355） | controls（n=473） |
| --- | --- | --- |
| 18-22 | 3 | 6 |
| 18-23 | 5 | 7 |
| 19-21 | 2 | 6 |
| 19-22 | 7 | 6 |
| 19-23 | 11 | 10 |
| 19-24 | 7 | 12 |
| 20-22 | 5 | 10 |
| 20-23 | 8 | 15 |
| 20-24 | 7 | 3 |
| 21-21 | 5 | 2 |
| 21-22 | 14 | 15 |
| 21-23 | 21 | 20 |
| 21-24 | 15 | 16 |
| 21-25 | 9 | 15 |
| 21-26 | 4 | 4 |
| 22-22 | 16 | 17 |
| 22-23 | 30 | 37 |
| 22-23.2 | 4 | 1 |
| 22-24 | 13 | 24 |
| 22-25 | 9 | 19 |
| 22-26 | 6 | 11 |
| 23-23 | 19 | 29 |
| 23-23.2 | 4 | 3 |
| 23-24 | 30 | 40 |
| 23-25 | 16 | 30 |
| 23-26 | 7 | 12 |
| 24-24 | 14 | 9 |
| 24-25 | 10 | 16 |
| 24-26 | 9 | 5 |
| 25-25 | 3 | 5 |
| χ2 | 25.90 |  |
| *p-value* | 0.631 |  |

*Note*: *Genotype frequency less than 1% in both groups were removed*
